# Biological characteristics and genomic analysis of a novel *Vibrio parahaemolyticus* phage phiTY18 isolated from the coastal water of Xiamen China

**DOI:** 10.3389/fcimb.2022.1035364

**Published:** 2022-10-21

**Authors:** Bo Liu, Tingyi Zheng, Rui Quan, Xinglong Jiang, Guixiang Tong, Xinxian Wei, Mao Lin

**Affiliations:** ^1^ Fisheries College, Jimei University, Engineering Research Center of the Modern Technology for Eel Industry, Ministry of Education, Xiamen, Fujian, China; ^2^ Guangxi Key Laboratory of Aquatic Genetic Breeding and Healthy Aquaculture, Guangxi Academy of Fishery Sciences, Nanning, China; ^3^ Key Laboratory of Healthy Mariculture for the East China Sea, Ministry of Agriculture and Rural Affairs, Xiamen, Fujian, China

**Keywords:** *Vibrio parahaemolyticus*, bacteriophage, biological characteristics, whole genome, myoviridae

## Abstract

*Vibrio parahaemolyticus* is a common pathogen usually controlled by antibiotics in mariculture. Notably, traditional antibiotic therapy is becoming less effective because of the emergence of bacterial resistance, hence new strategies need to be found to overcome this challenge. Bacteriophages, a class of viruses that lyse bacteria, can help us control drug-resistant bacteria. In this study, a novel *Vibrio parahaemolyticus* phage phiTY18 isolated from the coastal water of Xiamen was explored. Transmission electron microscopy showed that phiTY18 had an icosahedral head of 130.0 ± 1.2 nm diameter and a contractile tail of length of 66.7 ± 0.6 nm. The phage titer could reach 7.2×10^10^ PFU/mL at the optimal MOI (0.01). The phage phiTY18 had a degree of tolerance to heat and acid and base. At the temperature of 50°C (pH7.0, 1h) the survival phages reached 1.28×10^6^ PFU/mL, and at pH 5-9 (30°C, 1h), the survival phages was greater than 6.37×10^7^ PFU/mL Analysis of the phage one-step growth curve revealed that it had a latent period of 10min, a rise period of 10min, and an average burst size of the phage was 48 PFU/cell. Genome sequencing and analysis drew that phage phiTY18 had double-stranded DNA (191,500 bp) with 34.90% G+C content and contained 117 open reading frames (ORFs) and 24 tRNAs. Phylogenetic tree based on major capsid protein (*MCP*) revealed that phage phiTY18 (MW451250) was highly related to two *Vibrio* phages phiKT1024 (OM249648) and Va1 (MK387337). The NCBI alignment results showed that the nucleotide sequence identity was 97% and 93%, respectively. In addition, proteomic tree analysis indicated that phage phiTY18, phiKT1024, and Va1 were belong to the same virus sub-cluster within *Myoviridae*. This study provides a theoretical basis for understanding the genomic characteristics and the interaction between *Vibrio parahaemolyticus* phages and their host.

## Introduction

*Vibrio parahaemolyticus* is a common Gram-negative bacterium in aquaculture environment. *Vibrio parahaemolyticus* is short, rod-shaped, or arc-shaped, without spore and capsule structure, and with single polar flagella (Broberg et al., 2011). It is pathogenic to fish, shrimp and shellfish in the sea and can also infect the human body, therefore, it is a kind of zoonotic bacterium. Traditionally, farmers often used oxytetracycline, tetracycline, and quinolone antibiotics against *Vibrio parahaemolyticus* (Elmahdi et al., 2016). However, more resistant trains have developed because of antibiotic abuse, which poses a great threat to aquaculture.

In this study, we report a novel phage, phiTY18, isolated from seawater in Xiamen, Fujian, China, using *Vibrio parahaemolyticus* TY18 as the host. The study investigates the basic morphological, physiological, and biochemical characteristics and the whole genome of phiTY18 to provide a theoretical basis for future research or application at the control of *Vibrio parahaemolyticus.*

## Materials and methods

### Isolation and purification of the phage

The host strain, *Vibrio parahaemolyticus* TY18, was isolated and collected in our laboratory. Phage phiTY18 was isolated from seawater using the standard phage enrichment and double-layer agar methods (Ul Haq et al., 2012). The seawater samples were first mixed in a clean bucket, followed by the addition of the host, which was cultured to the logarithmic phase, and the subsequent overnight culturing of the mixture at 30°C. The mixture was then centrifuged at 10000×g/min, and the supernatant was filtered through a 0.22 μm filter membrane (Li et al., 2016). Phage plaques were displayed and observed using the double-layer agar methods (Vats et al., 1987; Zhang et al., 2017). A single plaque was selected and purified thrice, followed by placing the purified phages in SM buffer (100 mM NaCl, 8 mM MgSO_4_, 50 mM Tris-HCl (pH 7.5), 0.01% gelatin) for routine experiments or storage at 4°C. (Gao et al., 2017; Liu et al., 2017).

### Transmission electron microscopy

TEM was used to investigate the morphology of the phage particles. The purified phage solution (20 μL) with a titer of at least 10^10^ PFU/mL was first placed on a copper mesh to adsorb for 10 min. The sample was then negatively stained with 3% (w/v) phosphotungstate for 5 min. The phage morphology was subsequently observed by transmission electron microscope (JEOL Co., Tokyo, Japan) after natural drying at room temperature (Kwiatek et al., 2017).

### Optimal multiplicity of infection

The multiplicity of infection (MOI) refers to the ratio of the number of phages to that of hosts in the infection test. The phage and its host were mixed at the MOI of 1000, 100, 10, 1, 0.1, 0.01, 0.001, 0.0001, and 0.00001 after measuring their concentration. After incubation for 8 h at 30°C, the phage titer was determined using the double-layer agar methods. The ratio that can reproduce the highest titer of phage is the optimal MOI. (Abedon, 2016).

### One-step growth curve

The one-step growth curve was determined to measure the incubation period and the burst size of the phage. Phage was mixed with the host cultivated to exponential phase according to the optimal MOI (0.01). After adsorption at 30°C for 10 min, the supernatant was discarded after centrifugation at 10000×g for 2min. The residue was subsequently rinsed thrice using PBS buffer and resuspended with a 50 mL LB medium containing 3% NaCl. The resuspended residue was subsequently cultured in an incubator shaker set at 30°C. A sample was taken at 10 min intervals, up to 60 min, to detect the phage titer in real-time using the double-layer plate method. Phage burst size was calculated as the ratio of the phage titer at the end of burst phase to the concentration of host at the beginning of infection. The detections were done in three replicates, with the average value of the final result used to develop the growth curve.

### Thermal and pH stability assay

The phage content was cultured to 1×10^11^ PFU/mL for the experiment. The phage was incubated in a water bath set at 30°C, 40°C, 50°C, 60°C, 70°C, and 80°C for 60 min to determine its thermal stability. For the pH stability assay, the pH of the LB broth containing 3% NaCl was adjusted to 2, 4, 6, 7, 8, 10, and 12. The medium (900 μL) at different pH values and phage (100 μL) were mixed respectively, then incubated at 30 °C for 60 min. The double-layer agar methods was used to determine the phage titer (Wang et al., 2019).

### Host range of phage

Nine different strains of *Vibrio parahaemolyticus* were cultured to exponential stage, and LB plates were coated with 200 μl of bacterial solution. After the bacterial solution was air-dried, 20 μL phage phiTY18 was dropped on the coated plate. The plates were observed after 12 hours of incubation at 30 °C.

### Genome sequencing and bioinformatic analysis

The DNA of phage phiTY18 was extracted using the TIANamp Virus DNA/RNA Kit (TIANGEN) and was subjected to Illumina Hiseq second-generation map sequencing. The sequence reads were assembled using Canu, SPAdes, and HGAP. The assembled sequences with overlaps were made into a loop, followed by truncation of one side of the overlap sequence. The functions of the coding sequence were predicted using Glimmer, GeneMarkS, and Prodigal software. The sequences were compared and annotated based on references made to NR, Swiss-Prot, Pfam, EggNOG, GO, and KEGG databases. The genome was mapped using DNAPlotter (Carver et al., 2009; Liu et al., 2019).

### Comparative genomes and phylogenetic tree analysis

Phylogenetic trees was constructed based on the amino acid sequence of the major capsid protein (*MCP*) and the terminase large subunit (*TerL*) by using MEGA 7.0 to analyze the evolutionary relationship between phage phiTY18 and other phages (Kumar et al., 2008). A proteomic tree was drawn using the Viral Proteomic Tree server (https://www.genome.jp/viptree) (YNishimura et al., 2017). The whole genomes of phage phiTY18, phiKT1024 (OM249648), and Va1 (MK387337) were subsequently analyzed using Mauve software.

## Results

### Morphology of phage phiTY18

**Figure 1:**
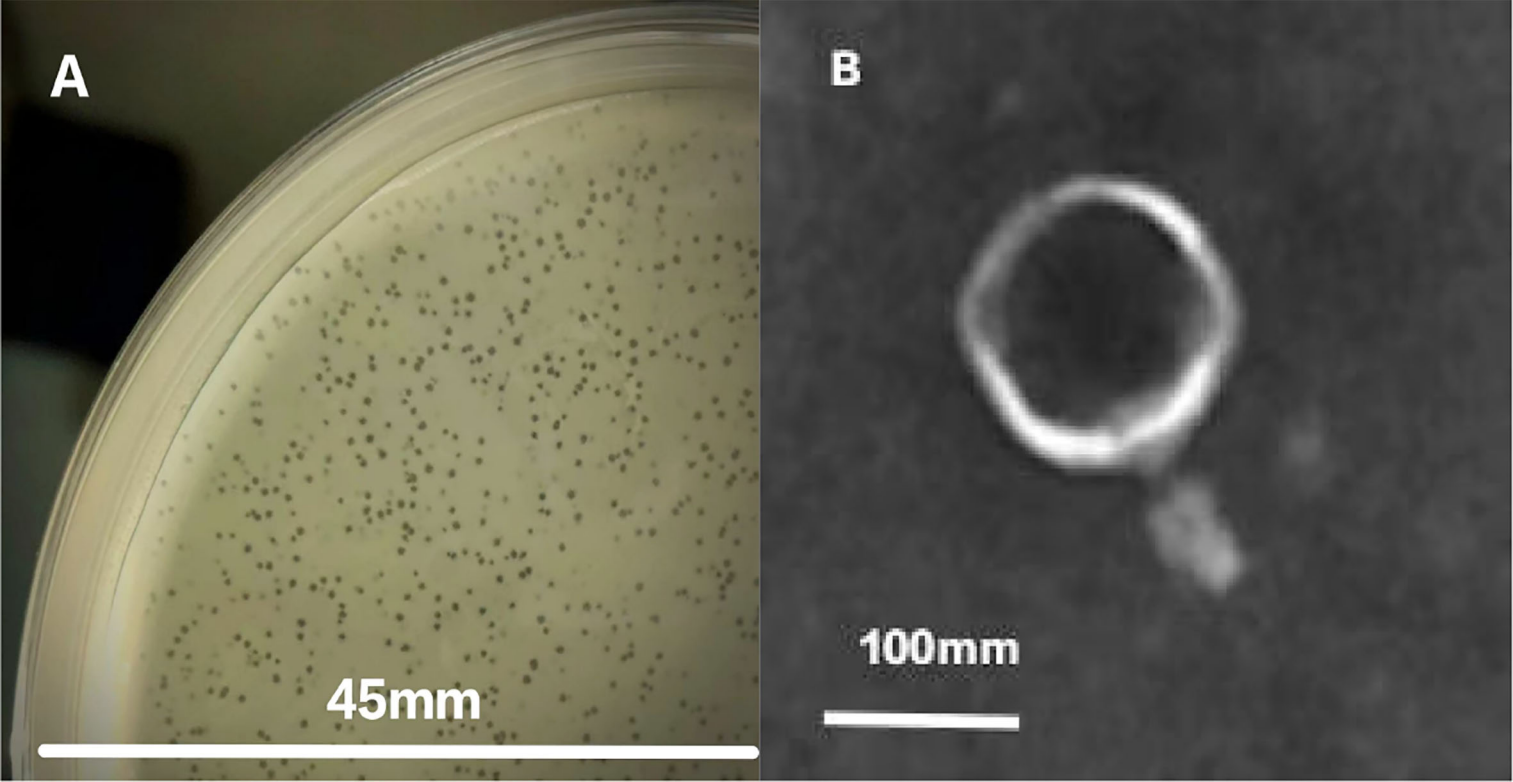
Plaque appearance **(A)** and virion morphology **(B)** of phage phiTY18.

Plaques formed by phages on a plate (**Figure 1A**). TEM micrographs show that phage phiTY18 has an icosahedral structure, with a head diameter of 130.0 ± 1.2 nm and a contractile tail length of 66.7 ± 0.6 nm (**Figure 1B**). Phage phiTY18 could be classified into *Myovirodae* based on the morphological characteristics, according to the classification and nomenclature standards of the virus proposed by the International Committee on Taxonomy of Viruses (ICTV, 2020).

### Optimal multiplicity of infection

The contents of phage were different under different MOI. The infection efficiency of phage was the highest and the maximum concentration of phage was 7.2×10^10^ PFU/mL at the optimal MOI (0.01) (**Table 1**).

**Table 1:** Multiplicity of infection of phage phiTY18.

MOI	Initial concentration		Final concentration of phage(PFU/mL)
	Phage(PFU/mL)	Host(CFU/mL)
1000	1×10^8^	10^5^	2.7×10^9^
100	1×10^8^	10^6^	2.4×10^10^
10	1×10^8^	10^7^	2.0×10^9^
1	1×10^8^	10^8^	5.4×10^9^
0.1	1×10^7^	10^8^	8.0×10^9^
**0.01**	**1**×**10^6^**	**10^8^**	**7.2**×**10** ^10^
0.001	1×10^5^	10^8^	3.2×10^9^
0.0001	1×10^4^	10^8^	1.4×10^9^
0.00001	1×10^3^	10^8^	2.2×10^8^

The bold words mean that the phage can reach the highest culture content at MOI.

### One-step growth curve

**Figure 2:**
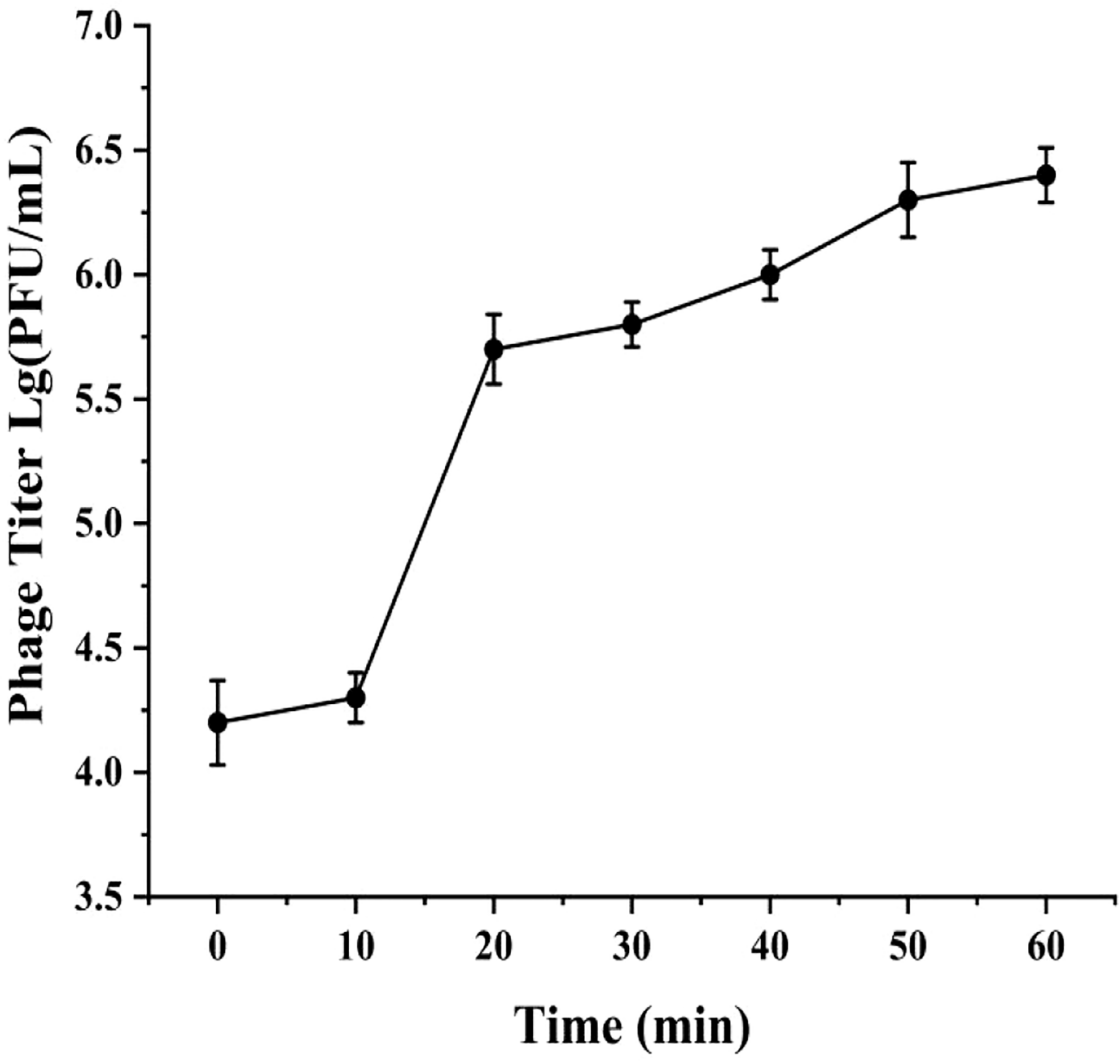
One-step growth curve of phage phiTY18. Each data point is the average of three independent experiments, and error bar represent standard deviations.

The one-step growth curve of phage phiTY18 showed that the latent period was approximately 10 min and the rise period was 10 min. The phage number gradually increased steadily and entered the plateau period after 20 min (**Figure 2**), and the average phage burst size was 48 PFU/cell.

### Thermal and pH stability

**Figure 3:**
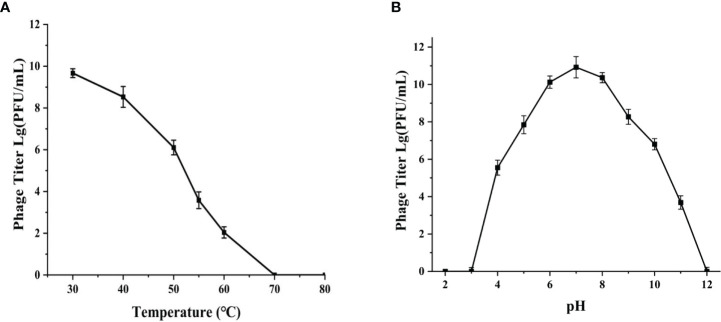
Thermal stability **(A)** and pH stability **(B)** of phage phiTY18. Each data point is the average of three independent experiments, and error bar represent standard deviations.

The phage phiTY18 still remained a high titer with above 1.28×10^6^ PFU/mL after incubating at 50°C for 1 h according to the **Figure 3A**. However, the phage activity gradually weakened with the increase in temperature and was completely lost at 70°C. The pH stability assay showed that the phage titer could be maintained above 6.37×10^7^ PFU/mL at pH 5 ~ 9 (**Figure 3B**). However, the phage completely lost its activity when the pH was 3 and 12. These findings suggested that strongly acidic and alkaline conditions affected the phage titer.

### Host range of phage

The host range of phage showed that phage phiTY18 could lyse three out of nine strains of *Vibrio parahaemolyticus* (**Table 2**).

**Table 2:** The result of Phage host spectrum.

bacteria	TY17	TY18	TY19	TY20	TY21	TY22	TY23	TY24	TY25
plaque	+	+	–	–	–	–	–	–	+

‘+’ indicates that bacteria can be lysed by phage phiTY18 to form plaques, while ‘-’ indicates that they cannot be.

### Genome analysis of phage phiTY18

**Figure 4:**
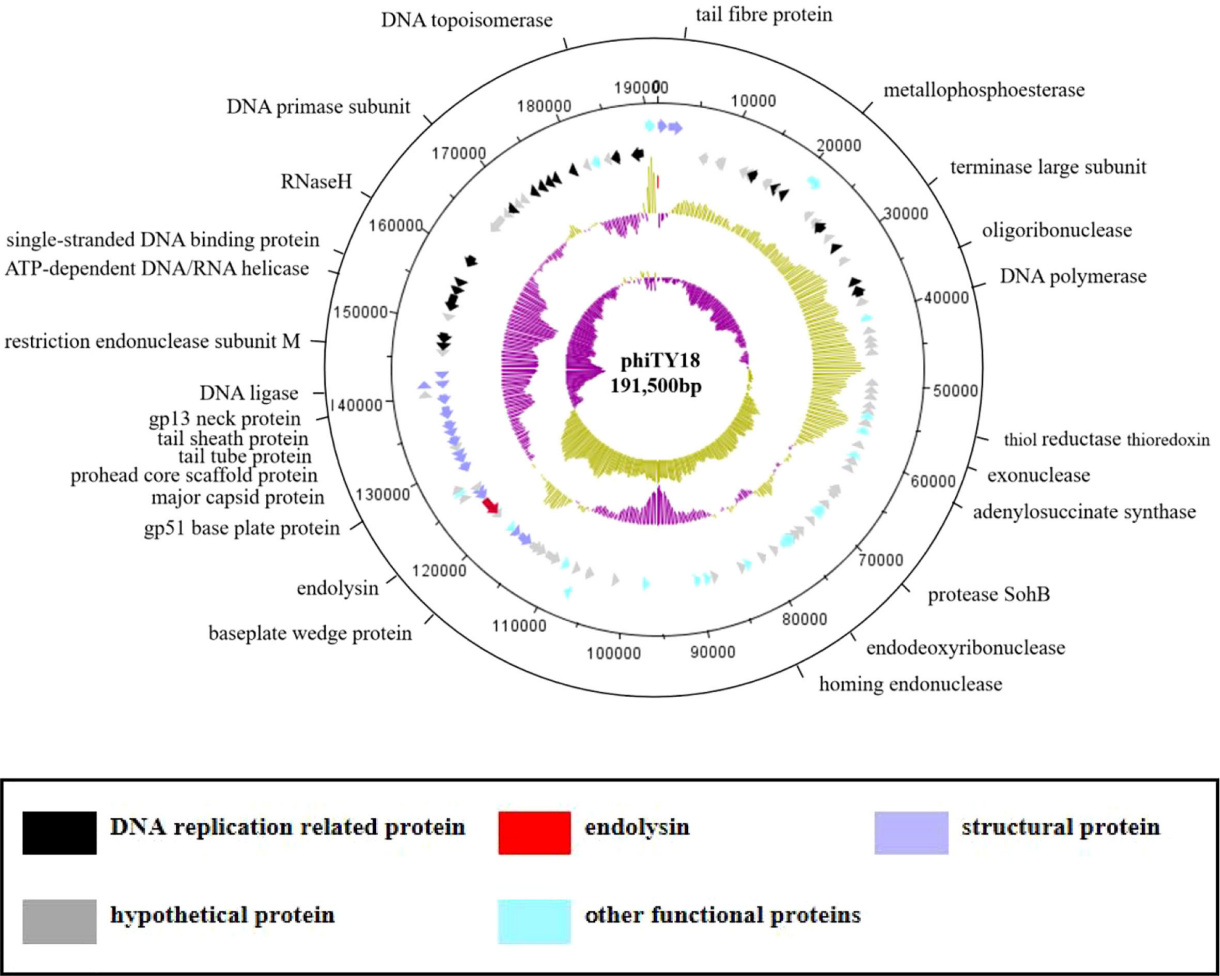
The genome map of bacteriophage phiTY18. The first and second circles from the outside to the inside are the CDS on the positive and negative strands. The different colors represent different gene functions. The third circle indicates the GC content. The inward and outward parts denote a lower GC content and higher GC content in the region compared to the average GC content of the whole genome, respectively. The fourth circle represents the GC-Skew value; GCskew>0 indicates the leading chain, while GCskew< 0 indicates the lag chain.

The genome sequencing results of phage phiTY18 revealed that the genome consisted of 191,500 bp, with a G+C% of 34.9% (**Figure 4**). Further analysis predicted that the phiTY18 genome contained 117 open reading frames (ORFs), including 60 (51%) functional ORFs and 57 (49%) hypothetical proteins with unknown functions (**Supplementary Table 1**). In addition, 24 tRNAs were predicted, but no rRNA was predicted. ORF1 was predicted to encode the tail fibrin, which typically interacts with bacteria as a receptor-binding protein (RBP) in bacteriophages of the *Myoviridae* (Sun et al., 2021). The bacteriophage tail has many protein structures which play a vital role in host cell recognition, adsorption, digestion, and phage genome injection. ORF91 encoded the head protein while ORF89 and ORF92 encoded the head portal protein and neck protein, respectively, thus forming a channel to export the genome (Zhang et al., 2017; Zhang et al., 2021). The proteins encoded by ORF18 and ORF101 in the genome replication module were predicted to be DNA helicases and thus could unlock the double helix structure of DNA and promote DNA replication. ORF25 was predicted to encode phage slide forceps, which assisted ORF22 and ORF23, which encoded two proteins with DNA polymerase activity, to replicate DNA. The protein encoded by ORF96 was predicted to be DNA ligase, which could catalyze the connection of double-stranded DNA and assist in DNA replication and recombination. In addition, ORF115 and ORF116 encoded topoisomerase, which could catalyze the breaking and binding of DNA strands. ORF104 encoded transcriptional regulators are essential for regulating the modification of protein synthesis during DNA replication. ORF71 encoded a phage tail lysozyme, a class of enzymes encoded by bacteriophages that can cleave bacteria. The tail lysozyme can attack the bacterial cell wall peptide polysaccharide layer, leading to the degradation of the cell wall layer and promoting the release of newly assembled virions (Trudil, 2015).

### Comparative genomes and phylogenetic tree analysis

**Figure 5:**
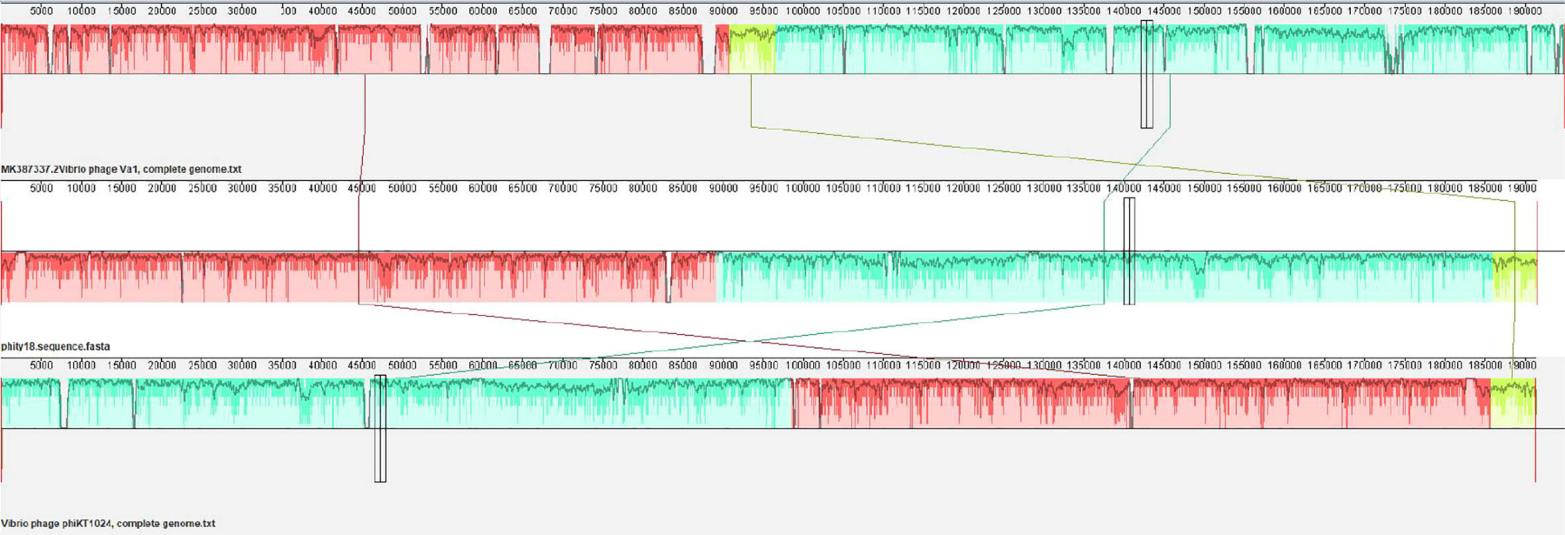
Collinear analysis of phage phiTY18 with *Vibrio* phage phiVa1 and phiKT1024. The collinearity analysis of three phages Va1, phiTY18 and phiKT1024 are shown. The same color indicates that this part of the sequence is similar.

**Figure 6:**
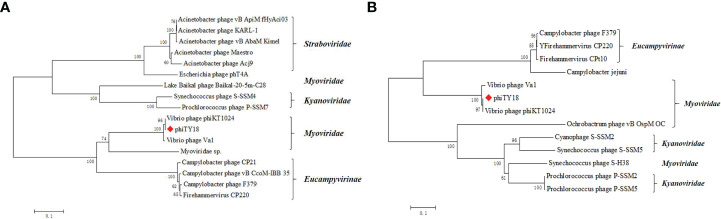
Phylogenetic tree based on major capsid protein (*MCP*) **(A)** and the terminase large subunit **(B)**. Phylogenetic trees were constructed by the neighbor-joining method with 1000 bootstrap replicates, following the ClustalW alignment of amino acid sequences using MEGA 7.0. The tree is drawn to scale, with branch lengths in the same units as those of the evolutionary distances used to infer the phylogenetic tree. The evolutionary distances were computed using the Poisson correction method and are in the units of the number of amino acid substitutions per site. The phage phiTY18 was labeled with a red diamond-shaped.

**Figure 7:**
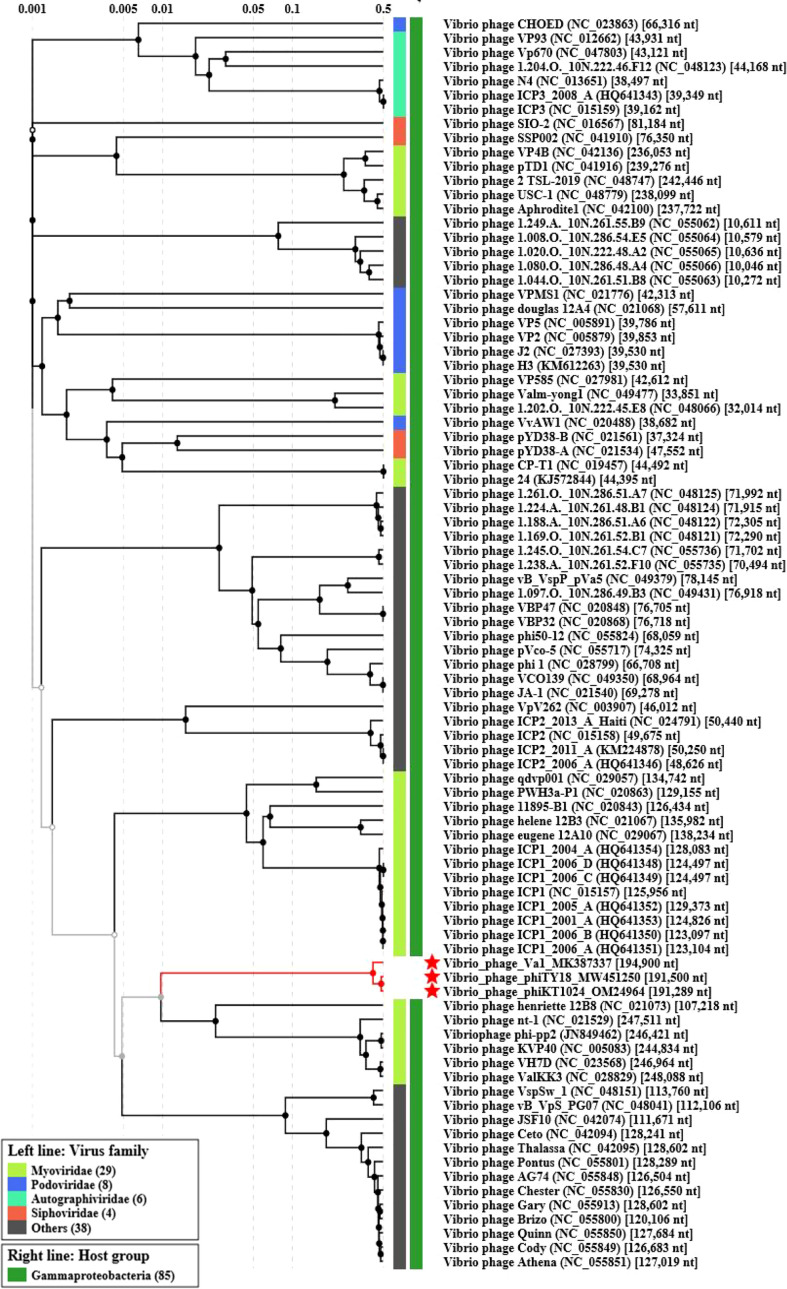
Proteomic tree analysis of *Vibrio* phage. Phages phiTY18, Va1, and phiKT1024 were labeled with a red star respectively.

The genome of phage phiTY18 shared high homology with two *Vibrio* phages, phiKT1024 (97%) and Va1 (93%), according to the blastn in NCBI. Genomic collinearity analysis further revealed their similarity (**Figure 5**). According to the phylogenetic tree constructed by *MCP* (**Figure 6A**) and *TerL* (**Figure 6B**), phage phiTY18 was found to be in the same branch with two similar *Vibrio* phages phiKT1024 and Val, and no other *Vibrio* phage was highly related to phiTY18. The VipTree server recorded 132 *Vibrio* phages, which were further divided into 9 different family (**Supplementary Table 2**). The results of proteomic tree (**Figure 7**) analysis also showed that these three phages were independent from a branch of *Myoviridae* and were distinguished from other phages in the database of VipTree, indicating that they probably represented a novel sub-cluster of *Myoviridae*.

## Discussion

In this study, a novel phage phiTY18 was isolated from seawater using *Vibrio parahaemolyticus* TY18 as a host. Morphologically, phage phiTY18 was a regular icosahedron, which had a head with a width of 130.0 ± 1.2 nm and a tail with a length of 66.7 ± 0.6 nm according to transmission electron microscopy. The phage burst size was 48 PFU/mL, and the phage titer was up to 7.2×10^10^ PFU/mL at the optimal MOI. Moreover, The one-step growth curve showed that compared with phage B23 and PH1, phage phiTY18 had a shorter incubation period (10min) and a faster lysis (10min) (Liu Z et al., 2017; Zhu et al., 2018). The results of temperature analysis showed that phage phiTY18 could maintain a titer above 1.28×10^6^ PFU/mL at 30-50 °C, and its activity decreased gradually with the increase in temperature. The optimal pH adaptation of phage phiTY18 was between 5 and 9 and was completely inactivated at pH ≦3 and ≧12 and the results are similar to the previous study. (Wang et al., 2019).

Genome sequencing results suggested that phiTY18 was dsDNA with a total length of 191,500bp. A comparison of the whole genome of phiTY18 with other known phages in the NCBI database revealed that it shared high homology with two *Vibrio* bacteriophages phiKT1024 (OM249648) and Va1 (MK387337), sharing nucleotide sequence identities of 97% and 93%, respectively. Among them, tail fibrin ORF1 and ORF2 related to phage host interaction share only 52.83% and 31.75% homology with tail fibrin of phage phiKT1024, which may be related to phage’s specific recognition of host through RBP (Zampara et al., 2020). However, the homology of other phage tail proteins, such as phage tail tubulin proteins (ORF84 and ORF85), can reach more than 98% (**Supplementary Table 1**). ORF117 is predicted to be a tail-completing protein with an important role in phage tail assembly (Pell et al., 2009). ORF80 is the putative major capsid protein among the structural proteins of phage phiTY18. The capsid protein acts as the shell of the phage and tightly encloses the genetic material of the phage. It is also commonly used in phage classification because its coding sequence is highly conserved (Bamford et al., 2005). Most potent phages have regions encoding replication-related enzymes in their genetic material (Li et al., 2012). Phage phiTY18 also had several ORFs related to DNA replication, regulation, and nucleic acid metabolism among the known functional ORFs. The replication-related proteins enable the genetic material of phages to enter the host cell and use the host enzyme system to replicate their genetic material and protein expression. Proteins in these DNA replication modules (ORF22, ORF23, ORF25, ORF101, ORF101, ORF109, ORF115) all had more than 90% homology compared with phage phiKT1024 (**Supplementary Table 1**). ORF58 (endodeoxyribonuclease) hydrolyzes DNA in host cells to provide DNA for phage synthesis (Kropinski et al., 2013).

Lysozyme can act on peptidoglycan on the bacterial cell wall and increase its solubility, causing cell rupture from the inside and the release of progeny phages (Fernandes and São-José, 2017; Etobayeva et al., 2018; Zhang et al., 2021). The lytic system of phage phiTY18 is encoded by gene of ORF71. The tail lysozyme encoded by ORF71 has 70% homology with the lysozyme of phage phiKT1024 (**Supplementary Table 1**), which indicates that the two phages may have the same lysis mode. Notably, phages have great limitations in practical application because of their high host specificity. In contrast, lyase has little host specificity. Numerous scholars have thus focused on phage lyase studies and the cloning and expression of lyase (Briers et al., 2014; Defraine et al., 2016).

Despite the numerous phage genome information in the current NCBI database, there are still many unknown areas in phage genome and functional annotation. This study details the biological characteristics and whole genome of a novel *Vibrio parahaemolyticus* phage phiTY18, providing an important theoretical basis for exploring the less known field of phages for use in practical applications.

## Data availability statement

The datasets presented in this study can be found in online repositories. The names of the repository/repositories and accession number(s) can be found below: https://www.ncbi.nlm.nih.gov/genbank/, MW451250.

## Author contributions

BL and ML drafted and revised the manuscript. All authors contributed to the article and approved the submitted version.

## Funding

This research was funded by the National Key R&D Program of China (2020YFD0900102), Xiamen Ocean and Fishery Development Special Fund (21CZP007HJ07) and Science and Technology Major Project of Xiamen (3502Z20221018).

## Conflict of interest

The authors declare that the research was conducted in the absence of any commercial or financial relationships that could be construed as a potential conflict of interest.

## Publisher’s note

All claims expressed in this article are solely those of the authors and do not necessarily represent those of their affiliated organizations, or those of the publisher, the editors and the reviewers. Any product that may be evaluated in this article, or claim that may be made by its manufacturer, is not guaranteed or endorsed by the publisher.
