# Supplementary material for: Biological characteristics and genomic analysis of a novel Vibrio parahaemolyticus phage phiTY18 isolated from the coastal water of Xiamen China
Source: Front Cell Infect Microbiol. 2022 Oct 21;12:1035364. doi: 10.3389/fcimb.2022.1035364 (PMC9633966; doi:10.3389/fcimb.2022.1035364)
Supplement: Supplementary file 1 [file Table_1.docx]

### Supplementary material

Table 1 Functional ORFs of phage phiTY18

| CDS ID | NR Description | Gene Length (bp) | Coverage (%) | Identity (%) |
| --- | --- | --- | --- | --- |
| ORF001 | tail fiber | 1326 | 52.83 | 33.70 |
| ORF002 | long tail fiber | 1893 | 31.75 | 39.30 |
| ORF008 | multifunctional tRNA nucleotidyl transferase | 1344 | 97.99 | 47.10 |
| ORF012 | RNA ligase and tail attachment protein | 894 | 98.65 | 42.50 |
| ORF014 | metallophosphoesterase | 582 | 92.23 | 39.80 |
| ORF015 | terminase large subunit | 1899 | 73.42 | 48.90 |
| ORF018 | ATP-dependent primase-helicase | 1557 | 84.94 | 43.50 |
| ORF020 | oligoribonuclease | 924 | 69.71 | 27.90 |
| ORF022 | DNA polymerase | 1131 | 98.40 | 42.20 |
| ORF023 | DNA polymerase | 1620 | 96.47 | 33.60 |
| ORF025 | sliding clamp loader | 933 | 98.39 | 42.50 |
| ORF035 | ATP-dependent Clp protease proteolytic subunit | 585 | 97.94 | 47.10 |
| ORF037 | thiol reductase thioredoxin | 300 | 95.96 | 46.40 |
| ORF039 | glycerol-3-phosphate cytidylyltransferase | 465 | 87.01 | 70.90 |
| ORF045 | exonuclease | 645 | 84.58 | 53.00 |
| ORF046 | adenylosuccinate synthase | 1137 | 84.92 | 41.60 |
| ORF051 | anaerobic ribonucleoside-triphosphate reductase activating | 444 | 95.92 | 44.80 |
| ORF052 | anaerobic ribonucleoside-triphosphate reductase | 1806 | 98.50 | 65.70 |
| ORF055 | protease SohB | 912 | 79.21 | 45.00 |
| ORF058 | endodeoxyribonuclease | 648 | 99.53 | 44.20 |
| ORF059 | homing endonuclease | 570 | 98.94 | 48.30 |
| ORF060 | glycoside hydrolase family 19 | 888 | 68.47 | 50.00 |
| ORF064 | gp49 EndoVII packaging and recombination endonuclease | 540 | 80.45 | 36.20 |
| ORF065 | gp55 T4-like sigma factor involved in late transcription | 831 | 57.61 | 44.40 |
| ORF069 | baseplate wedge | 1992 | 74.51 | 32.50 |
| ORF070 | base plate wedge subunit | 357 | 70.34 | 41.00 |
| ORF071 | baseplate hub and tail lysozyme | 1008 | 75.22 | 31.60 |
| ORF073 | endolysin | 2637 | 11.96 | 40.40 |
| ORF074 | gp51 base plate | 690 | 96.94 | 26.50 |
| ORF075 | baseplate hub subunit | 651 | 96.76 | 26.70 |
| ORF078 | thymidylate synthase | 864 | 100.00 | 69.90 |
| ORF080 | major capsid protein | 1368 | 94.29 | 51.80 |
| ORF081 | prohead core scaffold protein | 729 | 91.74 | 34.30 |
| ORF082 | gp21 T4-like prohead core scaffold and protease | 699 | 59.91 | 72.70 |
| ORF084 | gp19 tail tube protein | 582 | 99.48 | 48.70 |
| ORF085 | phage tail tube protein | 753 | 98.80 | 41.80 |
| ORF086 | gp19 tail tube protein | 777 | 92.25 | 30.90 |
| ORF087 | molecular chaperone GroEL | 1581 | 99.05 | 65.70 |
| ORF088 | phage tail sheath protein | 1947 | 70.99 | 50.00 |
| ORF089 | gp20 portal vertex protein of head | 1503 | 98.60 | 42.60 |
| ORF091 | head completion protein | 474 | 68.15 | 45.80 |
| ORF092 | gp13 neck protein | 729 | 96.69 | 31.40 |
| ORF095 | Ubiquinone biosynthesis O-methyltransferase | 606 | 84.58 | 31.40 |
| ORF096 | DNA ligase (T4 gp30-like) | 1449 | 69.09 | 36.30 |
| ORF098 | ribonucleotide-diphosphate reductase subunit alpha | 2346 | 95.77 | 54.70 |
| ORF099 | ribonucleoside-diphosphate reductase subunit beta | 1143 | 92.63 | 49.30 |
| ORF100 | restriction endonuclease subunit M | 807 | 63.81 | 33.50 |
| ORF101 | ATP-dependent DNA/RNA helicase | 1539 | 96.29 | 40.90 |
| ORF104 | transcriptional regulator | 408 | 71.85 | 57.70 |
| ORF107 | DNA primase subunit | 999 | 92.77 | 32.10 |
| ORF108 | RNaseH | 1068 | 92.11 | 41.00 |
| ORF109 | gp32 single-stranded DNA binding protein | 930 | 97.41 | 47.40 |
| ORF110 | sliding clamp | 705 | 94.44 | 32.10 |
| ORF111 | MreB-like ATPase protein | 963 | 98.44 | 52.50 |
| ORF113 | phosphate starvation protein PhoH | 1392 | 98.70 | 46.10 |
| ORF115 | gp52 DNA topoisomerase II | 1341 | 97.31 | 38.10 |
| ORF116 | gp39 topoisomerase II | 1803 | 76.33 | 36.60 |
| ORF117 | phage tail sheath completion protein | 1368 | 44.40 | 38.70 |

Note: The ORFs of the putative proteins are not shown in the table.

Table 2 Composition of all *Vibrio* phages in VipTree server

| Family of phage | Quantity of phage strains | Proportion of phages in total（%） |
| --- | --- | --- |
| Myoviridae | 40 | 30.3 |
| Demerecviridae | 19 | 14.4 |
| Autographiviridae | 18 | 13.6 |
| Schitoviridae | 17 | 12.9 |
| Podoviridae | 16 | 12.1 |
| Siphoviridae | 9 | 6.8 |
| Autolykiviridae | 5 | 3.8 |
| Zobellviridae | 5 | 3.8 |
| Ackermannviridae | 3 | 2.3 |
| total | 132 | 100 |
